# The molecular regulatory mechanisms of meiotic arrest and resumption in Oocyte development and maturation

**DOI:** 10.1186/s12958-023-01143-0

**Published:** 2023-10-02

**Authors:** Zhenle Pei, Ke Deng, Congjian Xu, Shuo Zhang

**Affiliations:** 1 Shanghai Ji Ai Genetics & IVF Institute, Shanghai Key Laboratory of Female Reproductive Endocrine Related Diseases, Obstetrics and Gynecology Hospital of Fudan University, Shanghai, 200011 China; 2 Department of Obstetrics and Gynecology of Shanghai Medical School, Fudan University, Shanghai, 200032 China

**Keywords:** Oocyte, Meiotic arrest, Meiotic resumption, cAMP, cGMP

## Abstract

In human female primordial germ cells, the transition from mitosis to meiosis begins from the fetal stage. In germ cells, meiosis is arrested at the diplotene stage of prophase in meiosis I (MI) after synapsis and recombination of homologous chromosomes, which cannot be segregated. Within the follicle, the maintenance of oocyte meiotic arrest is primarily attributed to high cytoplasmic concentrations of cyclic adenosine monophosphate (cAMP). Depending on the specific species, oocytes can remain arrested for extended periods of time, ranging from months to even years. During estrus phase in animals or the menstrual cycle in humans, the resumption of meiosis occurs in certain oocytes due to a surge of luteinizing hormone (LH) levels. Any factor interfering with this process may lead to impaired oocyte maturation, which in turn affects female reproductive function. Nevertheless, the precise molecular mechanisms underlying this phenomenon has not been systematically summarized yet. To provide a comprehensive understanding of the recently uncovered regulatory network involved in oocyte development and maturation, the progress of the cellular and molecular mechanisms of oocyte nuclear maturation including meiosis arrest and meiosis resumption is summarized. Additionally, the advancements in understanding the molecular cytoplasmic events occurring in oocytes, such as maternal mRNA degradation, posttranslational regulation, and organelle distribution associated with the quality of oocyte maturation, are reviewed. Therefore, understanding the pathways regulating oocyte meiotic arrest and resumption will provide detailed insight into female reproductive system and provide a theoretical basis for further research and potential approaches for novel disease treatments.

## Introduction

**Fig. 1:**
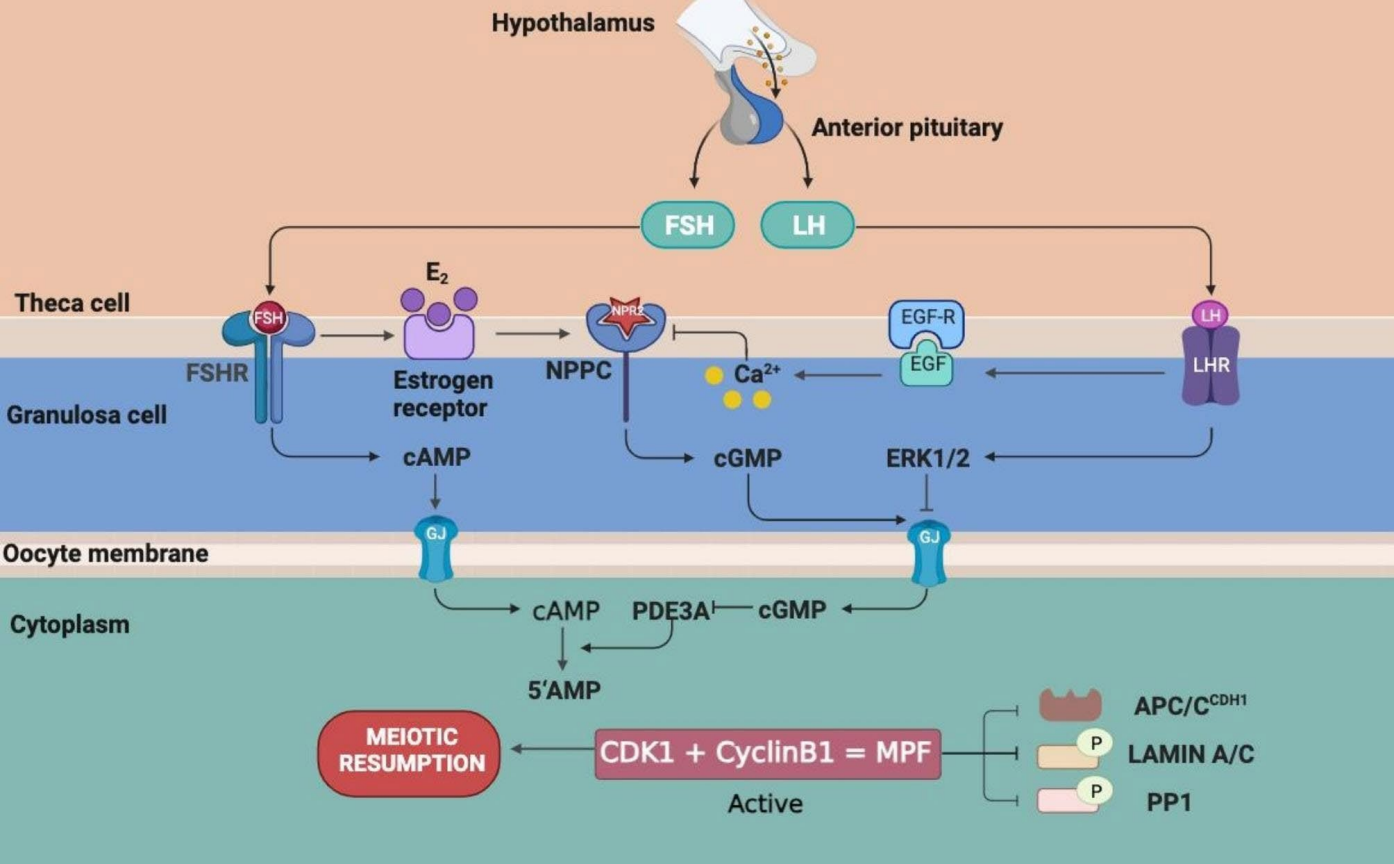
Schematic diagram of gonadotropin-induced meiosis I (MI) resumption. During the female menstrual cycle, GnRH secreted by the hypothalamus promotes the release of LH and FSH from the anterior pituitary. The LH surge shuts down follicular GJs to prevent the entry of cAMP and cGMP from GCs into oocytes. FSH upregulates cAMP levels in GCs and stimulates cumulus expansion. LH inhibits the NPPC-NPR2 signaling pathway by activating the EGFR-Ca^2+^ signaling pathway to downregulate cGMP levels in GCs, thereby reducing cGMP levels in oocytes and activating PDE3A-mediated hydrolysis of cAMP. Low cAMP levels activate MPF, which consists of CDK1 and cyclin B1, thereby promoting oocyte MI resumption. MPF not only phosphorylates and inactivates protein phosphatase 1 (PP1) but also phosphorylates APC/C to help maintain phosphorylation of other CDK1 substrates. Phosphorylation of lamin A/C leads to nuclear membrane rupture, which in turn promotes oocyte MI resumption

Elucidation of the signaling pathways coordinating oocyte meiotic arrest and resumption is a critical aspect of research on the female reproductive system. The development of follicles encompasses a series of sequential stages, which include the primordial, primary, secondary, preantral, antral, and pre-ovulatory follicle stages [1]. Notably, oocyte maturation differs from sperm maturation in several aspects. Firstly, female germ cells start meiosis I (MI) during embryonic development and are arrested at the diplotene stage of prophase of MI. Secondly, once females sexually mature, some arrested oocytes will resume MI, induced by a rapid increase in the luteinizing hormone (LH) secretion from the pituitary gland during estrus in animals or the menstrual cycle of women [2] (Fig. 1). Under normal circumstances, 10–20 arrested oocytes resume meiosis in each menstrual cycle, only one oocyte fully mature and is ovulated from the ovary [3]. During oocyte meiosis, cytokinesis is asymmetric, generating a polar body, with an extremely small amount of cytoplasm, and the oocyte, with most of the cytoplasm [4].

**Fig. 2:**
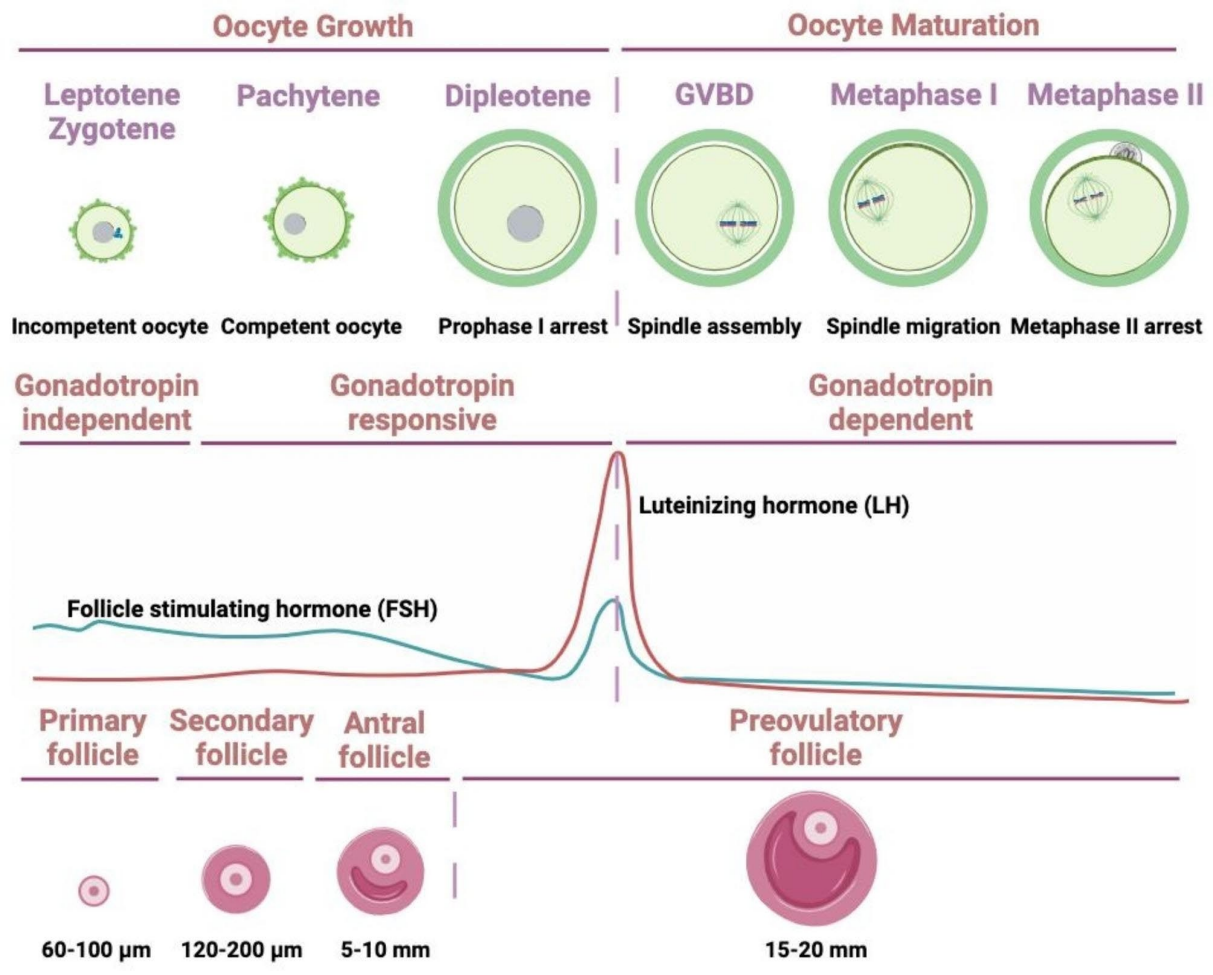
Schematic of the key stages of oocyte maturation and folliculogenesis. Upper panel: Oocyte growth, entry into meiosis and meiotic arrest. Meiotic maturation begins with GVBD, followed by the first meiotic division, divalent separation, extrusion of the first polar body, and arrest in the middle of the meiosis II. Oocytes ovulate at MII stage. Sister chromatids will separate after fertilization. Middle panel: Hormone levels in the corresponding states of oocyte maturation and follicular development. Growth of the oocyte and follicle can be classified into distinct stages. From primordial to secondary follicle development gonadotropin may be not needed. At this stage the follicular development may be termed gonadotropin-independent. Transition of the follicle from the preantral to early antral stage is primarily controlled by intraovarian regulators, the gonadotropin at this stage may not be required, termed as gonadotropin-responsive. The following growth past antral stage to the preovulatory stage may be gonadotropin-dependent. Lower panel: Follicle developmental stages and the sizes of follicles are indicated, with the oocyte eventually reentering meiosis and being released from the follicle

Each oocyte in an antral follicle contains a nucleus enclosed by nuclear membrane, referred to as germinal vesicle (GV). In the event of LH surge during the menstrual cycle, the oocytes in the antral follicles undergo a series of processes related to nuclear maturation, containing chromatin condensation and germinal vesicle breakdown (GVBD) (Fig. 2). After GVBD, the oocytes progress to MI metaphase. Subsequently, the first polar bodies (PB1) are extruded, while the oocytes complete MI. Then, the oocytes start meiosis II (MII) and are arrested at metaphase, awaiting fertilization. Meiosis is completed once oocytes are fertilized. The precise regulation of oocyte meiosis is an important guarantee for oocyte nuclear maturation, which is marked by the extrusion of PB1 [5]. The distribution and quantity of organelles, such as mitochondria, ribosomes, cortical granules, and endoplasmic reticulum, differ during the transition from GV to MII metaphase, the imbalanced distribution and functions of which influence oocyte cytoplasmic maturation [6].

Oocyte maturation from GV to MII includes a series of complex nuclear and cytoplasmic events that are prerequisites for oocyte development and maturation [7]. In mammals, including humans, multiple signaling pathways are considered critical for meiotic maturation. These pathways play a crucial role in regulating the maturation of oocytes and their interplay is essential for the production of high-quality oocytes. Any abnormalities in the various stages of these processes can result in ovulatory dysfunction, including conditions such as polycystic ovary syndrome (PCOS) that can lead to infertility [8]. However, the most advanced research progress on the regulatory mechanisms of oocyte meiosis has not been overviewed in the past decade. This review aims to examine the current research progress on the mechanisms involved in oocyte meiotic arrest and resumption in mammals, with the intention of establishing a foundation for further investigations into oocyte meiosis.

## Oocyte nuclear maturation

### Maintaining oocyte MI arrest

#### Elevated intra-oocyte cAMP level

In mammals, high cyclic adenosine monophosphate (cAMP) concentrations in oocytes are pivotal for maintaining meiotic arrest. Reduction in cAMP concentration triggers the resumption of meiosis [2, 9]. Under the circumstance that oocytes are separated from the antral follicles in vitro, intra-oocyte cAMP levels decrease, and MI resumes. In contrast, culturing oocytes with cAMP analogs, such as cAMP phosphodiesterase (PDE) inhibitors, prevents the meiotic maturation of oocytes [10]. High concentration of cAMP inactivates cyclin-dependent kinase 1 (CDK1) by activating protein kinase A (PKA), and inhibits the maturation promoting factor (MPF) composed of CDK1 and cyclin b1, thereby inducing GV arrest [11].

**Fig. 3:**
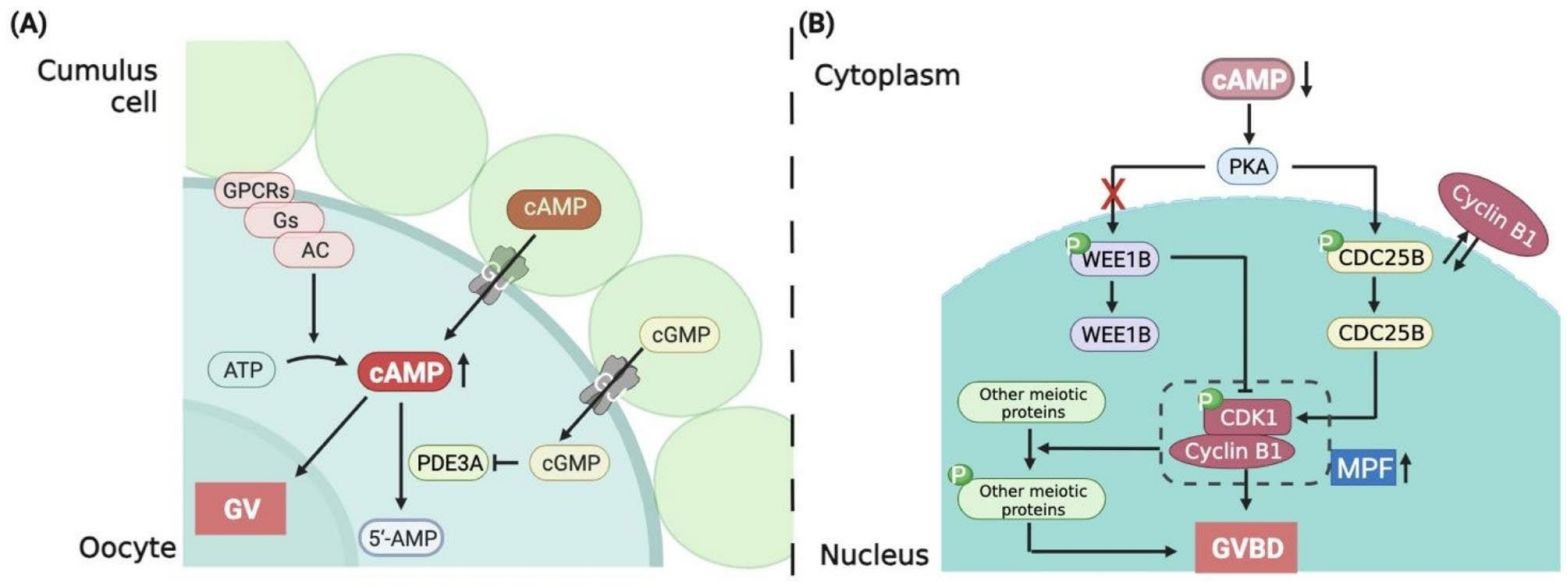
Mechanism of cAMP-regulated oocyte MI arrest and resumption. (**A**) Oocytes are arrested at prophase I by the following two mechanisms. (1) First, cAMP levels in oocytes are increased by exogenous cAMP from granulosa cells and cAMP generated in oocytes via the self-activated GPCRs-Gs-AC cascade. (2) Second, cGMP inhibits the hydrolytic activity of PDE3A, which in turn inhibits cAMP hydrolysis in oocytes. These two pathways work together to arrest oocytes at the diplotene stage of prophase of MI until the LH peak re-initiates meiosis. (**B**) Downregulation of cAMP levels in oocytes is induced by an increase in LH secretion. In the presence of low cAMP levels, Wee1B kinase remains inactive by activating PKA, while CDC25B phosphatase activity is increased, thereby activating MPF, which acts on the phosphorylation pathways of other meiosis-associated proteins to accelerate meiotic resumption

Phosphodiesterases (PDE) are important enzymes that regulate cAMP. Eleven different PDE isozymes (PDE1–11) have been identified in mammals. In contrast to PDE4D and PDE4B, which are localized in theca cells, cumulus granulosa cells (CGCs), and mural granulosa cells (MGCs), PDE3 is located exclusively in oocytes [12]. PDE3-specific inhibitors act on human, mouse, and bovine cumulus-oocyte-complexes (COCs) and denuded oocytes (DOs) to increase cAMP levels in oocytes and block meiosis progression in vitro [13]. In PDE3 knockout female mice, oocytes are arrested at GV phase, resulting in sterility. Inhibition of PDE3 increases cAMP levels and prevents maturation of cultured oocytes in COCs or DOs. In contrast, conditional knockout of G-protein coupled receptor 3 (GPR3) and PDE3A leads to oocyte maturation [14]. The cGMP synthesized in MGCs and CGCs enters the oocytes and inhibits the hydrolytic activity of PDE3A, thereby maintaining high cAMP levels in the oocytes [15]. Previous studies have reported that oocytes sustain meiotic arrest mainly via two pathways (Fig. 3). In the first pathway, cAMP levels in oocytes are up-regulated through both the import of exogenous cAMP from granulosa cells and the generation of endogenous cAMP via the self-activated GPCRs-stimulatory G protein-adenylyl cyclase (GPCRs-Gs-AC) cascade in oocytes [16]. In the second pathway, inhibition of PDE3A hydrolytic activity by cGMP inhibits cAMP hydrolysis in oocytes [17]. These two pathways work synergetically to arrest oocytes at the diplotene stage of prophase of MI until the surge in LH induces the re-initiation of meiosis. In PCOS patients, cAMP-dependent signal transduction is affected in theca cells and the increased expression of cAMP-GEFII (a cAMP sensor) is one mechanism for the differential expression of normal and PCOS theca cell gene [18]. In endometrial stromal cells, the basal and cAMP-driven PKA pathway activation was significantly lower in PCOS patients compared with controls [19].

#### Intercellular communication via gap junctions (GJs)

GJs play a vital role in facilitating communication between granulosa cells within follicles and with the oocytes themselves, and they are composed of connexin (Cx) family proteins [20]. Typically, GJs are open, allowing the transfer of molecules smaller than 1000 Da to the adjacent cells. Nutrients that support oocyte metabolism, including amino acids and glucose; ions that regulate oocyte pH; cGMP and cAMP, which are required to keep meiotic arrest in oocytes, can be delivered to oocytes via GJs [21]. However, during the peak concentration of LH, the translation of connexin43 (Cx43) is inhibited, leading to the disintegration of GJs and the subsequent disruption of communication between oocytes and granulosa cells. This causes decreased cAMP and cGMP concentrations in oocytes, leading to meiotic resumption [22]. Cx43 proves to be the major connexin in the GJs of CGCs [23]. In the absence of Cx43, the oocytes become stunted, given the diminished responsiveness of granulosa cells to growth differentiation factor 9 (GDF9), an oocyte-derived paracrine factor, facilitating the proliferation of granulosa cells [24]. Mitogen-activated protein kinase (MAPK) signaling associated with the LH surge may contribute to the induction of Cx43 phosphorylation in CGCs during the process of meiotic resumption [25]. Cx37, which is localized in the GJs on oocytes, is responsible for oocyte-granulosa cell metabolic coupling and is regulated via the signal-regulated kinase 1/2 (ERK1/2) signaling during the LH surge [26]. Knockout of gap junction protein alpha 4 (GJA4), which encodes Cx37, causes the GJs between oocytes and granulosa cells to disappear [27], leading to oocyte growth arrest, loss of meiosis resumption, degeneration, and ultimately apoptosis [28]. It remains unclear whether the other Cxs, such as Cx26, Cx30, Cx30, Cx32, as well as Cx40 assemble into intrafollicular GJs [29].

#### Import of exogenous cAMP

The cAMP produced by the follicular cells surrounding the oocyte is transported into oocytes via GJs. The addition of follicle-stimulating hormone (FSH) to in vitro cultures of mouse COCs delays MI resumption in the oocyte by approximately 3 h, whereas the addition of FSH to a culture of DOs does not increase cAMP and delays MI resumption [30]. Studies have reported that FSH induces an increase in cAMP in CGCs and stimulates cumulus expansion through the MAPK pathway [31]. The neurohormone 5-hydroxytryptamine also increases Ca^2+^ and cAMP levels in CGCs [32]. A high concentration of cAMP in CGCs induces the production of a large amount of progesterone, which leads to decreased GJs and a subsequent decrease in cAMP in porcine oocytes, thereby accelerating GVBD [33]. Therefore, cAMP in CGCs is associated with the maintenance of MI arrest and also contributes to the initiation of MI recovery [34].

#### Endogenous cAMP production

Oocyte itself can produce cAMP by expressing the proteins in the GPCRs-Gs-AC cascade, which are required for cAMP production, and the elimination of any factor in this pathway leads to oocyte maturation [33]. Inhibition of the Gs-G proteins in oocytes can also lead to meiotic resumption [35]. Mouse oocytes lacking adenylyl cyclase 3 (AC3) are unable to maintain meiotic arrest [36]. Spontaneous meiotic resumption at the early antral follicle stage in GPR3 knockout mouse oocytes is reversed by injecting GPR3 messenger ribonucleic acid, sustaining a role of endogenous cAMP production in maintaining arrest [37]. In vitro studies have revealed that inactivation of Gs or downregulation of GPR3 can restore meiosis in some PDE3A-deficient oocytes [16, 38]. Hybridization of PDE3A^−/−^ mice and GPR3^−/−^ mice results in partial restoration of female fertility. GPR3 is epistatic to PDE3A, and oocyte MI arrest in PDE3A-deficient mice is dependent on GPR3 activity. The increase of cAMP after PDE3A deletion was not detected in double mutant oocytes, which confirmed the function of GPR3 in regulating oocyte cAMP upstream of PDE3A [35].

#### Transfer of cyclic GMP

Cyclic GMP (cGMP), which is imported into oocytes from granulosa cells through GJs, is a water-soluble second messenger that inhibits PDE3A activity. Guanylate cyclase (GC) in MGCs and CGCs converts guanosine triphosphate (GTP) to cGMP, which enters oocytes to inhibit PDE3A activity, thereby maintaining high cAMP levels and MI arrest [39]. Follicular imaging suggests that cGMP concentrations are homogeneous in MGCs, CGCs, and oocytes. Mechanical release of oocytes from the follicles and disruption of the GJs between granulosa cells and oocytes can reduce cGMP levels in the oocytes, indicating that cGMP diffuses through GJs [40].

The natriuretic peptide receptors (NPRs) in mammalian follicles play a critical role in regulating oocyte meiotic arrest. NPR2, a major member of the NPR family, is mainly located on CGCs, whereas its ligand, known as natriuretic peptide precursor C (NPPC), is expressed in MGCs in mice [41], humans [42], and pigs [43]. Natriuretic peptide precursor C inhibits spontaneous GVBD in COCs but exerts little inhibitory effect on DOs. Mice with mutations in NPPC and NPR2 undergo premature meiotic resumption, resulting in disorganized chromosomes and cytoplasmic fragments in preovulatory oocytes [44]. These findings strongly suggested that oocyte meiotic arrest is mediated by the NPPC/NPR2 signaling pathway in follicular somatic cells. Furthermore, follicular stimulating hormone maintains high NPPC/NPR2 levels, and estrogen interacts with FSH to promote NPPC expression in granulosa cells. A sustained over-activated CNP/NPR2 system in the follicles of PCOS mice leads to accumulating cGMP and cAMP in CGCs [42]. In addition, researchers have reported that LH upregulated the expression of the NPPC/NPR2 system in ovarian granulosa cells from mice with PCOS and granulosa cell lines cultured in vitro through the binding of androgens and estrogens to their respective receptors, and inhibited MI resumption by elevating cGMP levels in CGCs [45, 46]. Therefore, the NPPC/NPR2 pathway might potentially be involved the mechanism of ovulation impairment in PCOS. Moreover, Yang et al. demonstrated that transforming growth factor (TGF)-β regulates NPPC expression in MGCs and oocyte maturation [47]. The mechanisms underlying oocyte meiotic arrest in mammals are summarized in Fig. 3A.

##### Activation of IMPDH in CGCs proves its involvement in oocyte meiotic arrest

In mouse ovaries, IMPDH maintains oocyte meiotic arrest by providing GTP substrate for NPPC/NPR2 system in CGCs to produce cGMP, which is essential for maintaining oocyte-follicular development synchronization. IMPDH plays a catalytic role in GTP production [48]. The expression of IMPDH in CGCs is promoted by oocyte-derived paracrine factors, and IMPDH-specific inhibitors can lead to oocyte meiotic resumption, indicating that fine regulation of IMPDH expression is essential for the control of oocyte meiosis. However, how IMPDH is accordingly changed with the induction of oocyte meiotic resumption remains unclear [49, 50]. Additionally, IMPDH also maintains the concentration of hypoxanthine (HX) in follicular fluid which inhibits PDE activity in oocytes, increasing intracellular cAMP levels to maintain meiotic arrest [51].

### The initiation of oocyte MI resumption

#### Promotion of MI resumption by gonadotropins

**Fig. 4:**
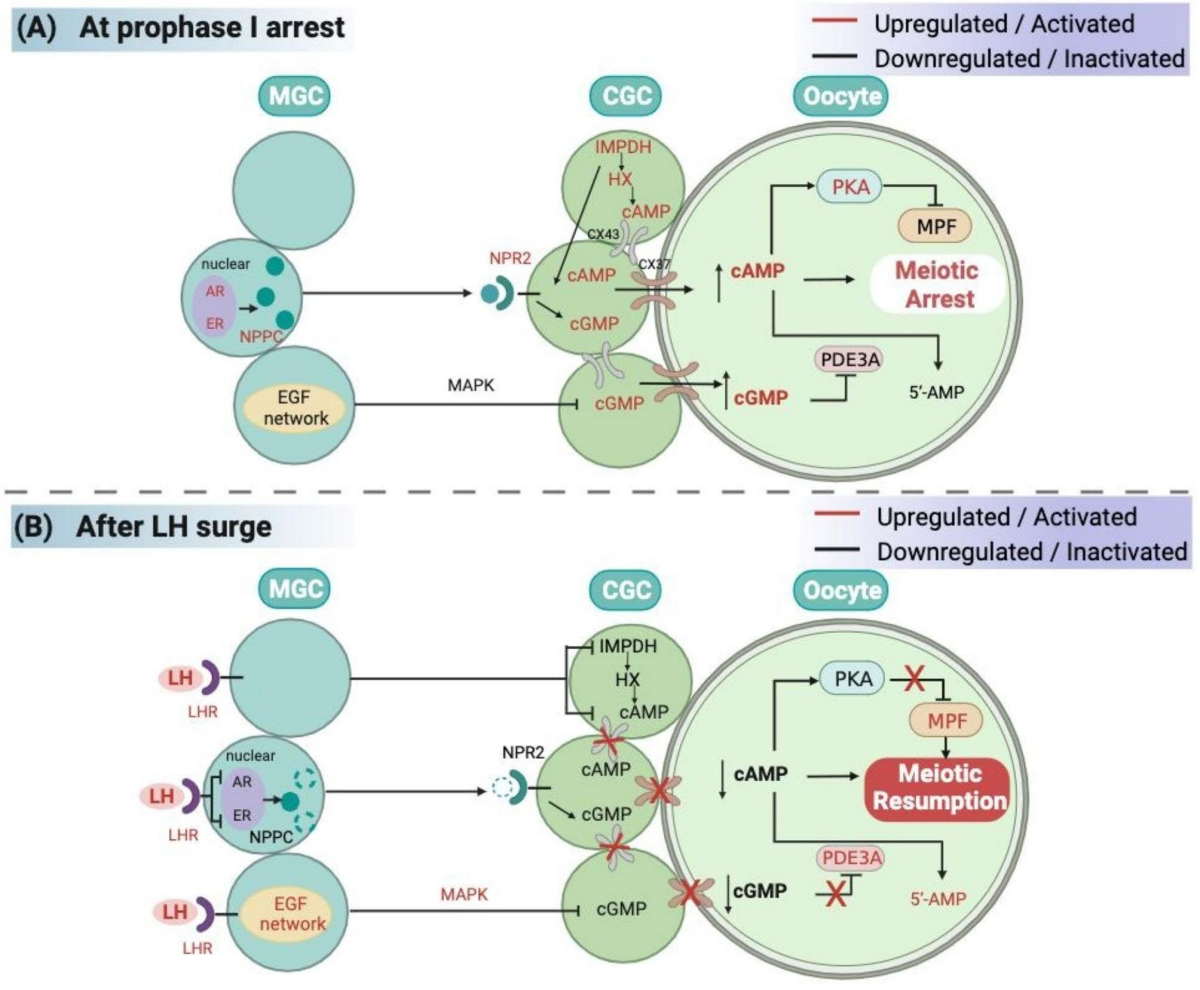
Schematic diagram of the mechanisms by which meiosis-related genes regulate oocyte MI arrest and resumption. Upregulated genes are shown in red, and downregulated genes are shown in black. (**A**) Maintenance of oocyte arrest at prophase I requires high cAMP levels. CGCs produce NPPC, and the presence of NPR2 in MGCs stimulates the production of cGMP, which enters oocytes through Cx37 in GJs and prevents PDE3A from hydrolyzing cAMP. cAMP activates PKA, leading to the inactivation of MPF and maintenance of MI arrest. (**B**) A sharp increase in LH secretion inhibits AR and ER to reduce the transcription and production of NPPC and increases EGF expression to activate EGFR signaling and increase calcium levels in CGCs to further inactivate NPR2. LH also causes follicular GJs to shut down, preventing cGMP from entering oocytes. Low cAMP and PKA levels in turn increase MPF levels, thereby promoting meiotic resumption

The main gonadotropins associated with the signals in follicular granulosa cells and MI resumption are LH and FSH. According to the hypothalamic-pituitary-ovarian axis feedback theory, LH initiates oocyte GVBD through positive feedback regulation in response to a peak in estrogen levels (Fig. 4). Luteinizing hormone binds to LH receptor (LHR), which causes a decrease in cGMP levels in granulosa cells by downregulating the NPPC/NPR2 system, thereby reducing the diffusion of cGMP within oocytes [39] via a mechanism that may be related to the ability of LH to significantly reduce the levels of androgen receptor (AR) and estrogen receptor (ER) [52]. However, how LH and FSH specifically regulate AR and ER remains unclear. LH shuts down the GJs between CGCs and oocytes, reducing intra-oocyte cAMP levels [53] and up-regulating epidermal growth factor (EGF) network in MGCs/CGCs. A reduction in cAMP within oocytes triggers the activation of MPF, initiating chromosome segregation and GVBD [54].

FSH can promote follicular growth and development, granulosa cell proliferation, and induce granulosa cells to generate LH receptors. The molecular association of FSH signaling with cumulus cell differentiation and cumulus-oocyte communication as well as transzonal projection dynamics may influence oocyte meiotic resumption. In addition, FSH also regulates cytoskeletal and spindle dynamics, metabolism, and DNA integrity in the oocyte through transzonal projections [55].

#### Activation of MPF

In mammals, MPF is a heterodimer composed of the catalytic subunit CDK1 and the regulatory subunit cyclin B. CDK1 phosphorylates serine and threonine residues on the premise that CDK1 is bound by cyclin B [56]. The timing of CDK1 activation is significant to the initiation of oocyte meiosis. Elevated cAMP levels activate PKA, which phosphorylates and activates the nuclear kinase Wee1B/Myt1. PKA also inactivates cell division cycle 25B (CDC25B), which is an activator of CDK1. Inactivated CDC25B loses its ability to dephosphorylate serine and threonine residues on CDK1, thereby maintaining MPF in an inactivate state. Studies have reported that infertility in CDC25B-deficient female mice resulted from permanent meiotic arrest caused by consistently low MPF levels [57]. Additionally, some epigenetic regulatory molecules, such as lysine (K)-specific demethylase 1 A (KDM1A), also regulate CDC25B expression to maintain MI arrest. Conditional knockout of KDM1A in oocytes resulted in early meiotic resumption and spindle and chromosomal abnormalities [58]. The surge of LH before ovulation reduces the level of cAMP, which no longer activate WEE1B, inactivate CDC25B, dephosphorylate CDK1 and becomes catalytic active. The active CDK1-cyclin B1 complex inactivates protein phosphatase 1 (PP1) phosphorylation, which facilitates to maintaining the phosphorylation status of other CDK1 substrates [59]. Phosphorylation of laminin A/C leads to nuclear membrane rupture. The active CDK1 can also phosphorylate other meiotic proteins that associated with oocyte GVBD [60].

Cyclin B1 is continuously degraded by anaphase promoting complex/cyclosome (APC/C), which is a multimeric E3 ubiquitin ligase [61]. Reduction in cyclin B1 activity induces a non-activated state of MPF, ultimately arresting oocytes at MI. Cadherin 1 is an activator of APC/C [62]. Therefore, degradation of cyclin B leads to both the inactivation of CDK1 phosphorylation and a decrease in MPF activity, which together arrest oocytes at MI [63]. Recently, some cyclin B1-deficient oocytes were shown to resume and complete MI via a mechanism in which cyclin B2 compensates for the CDK1-activation activity of cyclin B1 during the oocyte meiotic G2/M transition [43, 64].

#### Growth factor-related proteins

Growth factor-related proteins include growth hormone (GH), EGF, insulin-like growth factor (IGF), and TGF [65]. GH secreted by ovarian tissues directly binds to the GH receptor on the ovary. In older women, GH increases the expression levels of follicle-stimulating hormone receptor (FSHR) and luteinizing hormone receptor (LHR) in granulosa cells, as well as the density of FSHR, bone morphogenetic protein receptor type 1B (BMPR1B), LHR, and GH receptor (GHR) in granulosa cells. This leads to improved ovarian responsiveness and a significant increase in pregnancy rates [66]. Meiotic resumption is also promoted by GH via promoting the production and the uniform distribution of mitochondria in mouse oocytes [67, 68].

EGFs, a protein family responsive to LH signaling, play a crucial role in oocyte maturation. Various epidermal-like growth factors, including amphiregulin (AREG), and β-cellulin (BTC), are present in and secreted by CGCs and MGCs through autocrine as well as paracrine mechanisms. These growth factors function via their specific EGF receptors (EGFRs) [69]. Studies have revealed that mouse oocytes with granulosa cell-specific EGFR deficiency fail to resume meiosis [70]. LH stimulates EGF secretion by MGCs to activate EGFR signaling in granulosa cells to activate PDE5 [71, 72]. In GCs, histone deacetylase 3 (HDAC3) is a negative regulator of EGF expression prior to the LH surge [73] and is recruited to the AREG promoter by transcription factors, such as forkhead box protein O1 (FOXO1), to inhibit AREG expression [46]. With the LH surge, HDAC3 levels decrease, while acetylation of histone H3 Lysine 14 increases, allowing the transcription factor specificity protein 1 to bind to AREG promoter to induce its transcription [74, 75]. The EGFR signaling pathway also activates phospholipase Cγ, leading to an increase in calcium levels [76]. Elevated calcium concentrations in granulosa cells result in the inactivation of NPR2, reducing the binding between NPR2 and NPPC. Therefore, reduced cGMP levels are closely associated with MI resumption. However, the specific molecular mechanism underlying LH regulation of EGF remains unclear [77].

### Oocyte cytoplasmic maturation

#### Expression and degradation of maternal mRNA

Upon initiation of meiotic resumption in oocytes, transcription terminates to chromatin condensation, and maternal mRNAs are degraded and gradually consumed [78, 79]. However, from anaphase onwards, *de novo* mRNA synthesis commences, and a stringent mechanism for mRNA stabilization operates during the GV phase of oocytes. A key player in oocyte maturation is cytoplasmic polyadenylation of the 3’ untranslated region, which not only influences mRNA stability but also activates translation [80]. During the transition from the GV to MII stage in mouse oocytes, there is a highly selective degradation of mRNAs related to oxidative phosphorylation, while protein synthesis is tightly regulated [81]. Selective degradation of maternal mRNA transcripts is a prerequisite for zygotic genome activation [82]. Regulation of the translation and degradation of maternal mRNA primarily takes place in mature oocytes instead of fertilized ova, and the mechanisms are critical for completing the maternal-to-zygote transformation (MZT) [83].

#### Post-transcriptional and translational regulation

**Fig. 5:**
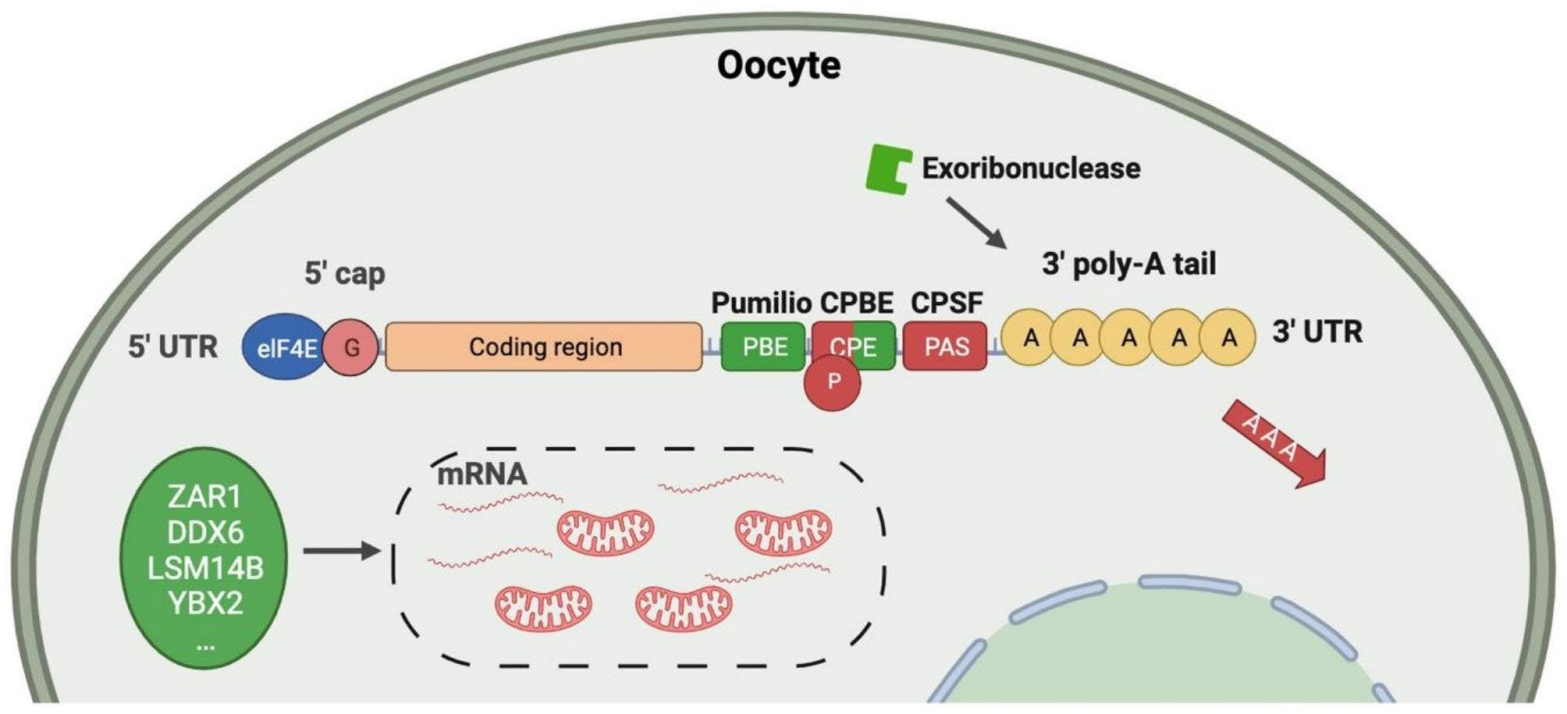
Schematic diagram of the key 3′-UTR elements and RNA binding proteins (RBPs) regulating poly(A) tails in oocytes. The 3′-UTR elements and RBPs mediating polyadenylation and translational activation are shown in red, and the 3′-UTR elements and RBPs mediating deadenylation and translational repression are shown in green. As shown in the dotted box is mitochondria-associated ribonucleoprotein domain associated with several recently discovered functional RBPs.

Embryonic genome activation (EGA) transcription is in effect silenced from GV to post-fertilization [84]. Therefore, mRNA transcripts produced by oocytes are required to meet their protein requirements during meiosis, fertilization, and MZT. After meiosis resumes, the control of gene expression transfers from transcriptional control in the nucleus to translational regulation in the cytoplasm. Maternal RNA binding proteins (RBPs) function as translational regulators, contributing significantly to oocyte maturation and early embryonic development (Fig. 5) [85]. A portion of the mRNAs transcribed during oocyte growth (30–45%) is translationally repressed prior to meiotic maturation or fertilization [86]. These stored mRNAs are usually adenylated upon export from the nucleus, leaving a 20–40 residue poly (A) tail that is detrimental for translation on the grounds that eukaryotic translation initiation factor 4G (eIF4G) is unable to bind to poly (A)-binding protein (PABP) [87]. Binding to specific RBPs facilitate mRNAs maintaining in a stable adenylated state until they are de-repressed when translation is required, and the poly (A) tails are restored. This process is usually mediated by phosphorylation of RBPs, as a part of signaling cascade. Recently, studies had established that RBPs suchas YBX2 and ZAR1 are required for maternal mRNA storage in full-grown mouse oocytes. ZAR1 promotes mitochondria-associated ribonucleoprotein domain assembly and coalescence into clusters [88].

The most well-studied mechanism involves CPE-binding proteins (CPEBs) and cytoplasmic polyadenylation elements (CPE) located at the 3′-UTRs of mRNAs [89, 90]. CPEB1 is expressed in mouse oocytes until MI phase and controls the translation of MAPK, kinase c-Mos, and cyclin B1, affecting meiosis progression [91]. Another mechanism involves Pumilio (PUM) protein. PUM1/2 is thought to mediate translation repression by interacting with the carbon catabolite repression-negative on tata-less (CCR4-NOT) adenylase complex [92, 93]. Evidence regarding the participation of other RBPs in oocyte mRNA regulation, like Y-box binding protein 2 (YBX2) and deleted in azoospermia-like (DAZL), remain unclear [94, 95]. However, YBX2 is highly expressed in mouse oocytes, accounting for approximately 2% of the total protein [82], and deletion of YBX2 in mouse GV oocytes resulted in abnormal oocyte growth, decreased mRNA stability, an inability to terminate transcription, and significant disruption of the transcriptome. DAZL is an important mRNA that regulates spindle assembly, cell meiosis, as well as mRNA degradation [94, 96]. Further research on additional mechanisms by which oocyte-specific RBPs regulate mRNA and genome stability is warranted.

#### Organelle distribution

Oocyte cytoplasmic maturation includes the timely maturation (the proper structure and distribution) of multiple organelles, particularly mitochondria, cortical granules, cytoskeleton, and endoplasmic reticulum [97]. Cortical granules, which are membranous organelles originated from the Golgi complex, are present in the cortex of unfertilized oocytes and play a crucial role in fertilization by undergoing exocytosis after fertilization, leading to polyspermy block in the zona pellucida in mammals [98]. Mitochondria are critical for supplying energy to oocytes with adenosine triphosphate (ATP) [99]. Low oocyte quality, caused by failures in meiotic chromosome segregation, maturation, and fertilization is associated with mitochondria dysfunction, including abnormal rearrangement, low numbers, and low ATP levels [100]. Additionally, endoplasmic reticulum is capable of storing and releasing free Ca^2+^ in the cytoplasm, which is essential for the calcium response during fertilization. The cytoskeleton, primarily composed of microtubules and filaments, regulates spindle reorganization with the microfilament network during meiosis. Any disruption of the microtubules or microfilaments may lead to failed chromosome movement and segregation, resulting in metaphase arrest [101].

## Conclusions

This review provides a comprehensive summary of current research on cellular and molecular events which occur during oocyte meiosis, specifically focusing on the mechanisms related to MI arrest and resumption. The understanding of the dynamics and functions of gap junctions (GJs), the only means of communication between oocytes and their follicular environment, remains incomplete. The exact mechanism by which LH triggers MI in oocytes, whether LH through its receptor or indirectly via EGFR signaling pathway, is still uncertain. Further investigation is needed to explore the role of extracellular vesicles in regulating the differentiation of follicular somatic cells and oocytes [102]. Future research should also concentrate on studying epigenetic changes. post-translational modifications and the roles of non-coding RNAs in meiosis [103]. A more detailed understanding of key events in oogenesis can be achieved by utilizing advanced imaging techniques and high-throughput analyses, such as single-cell-based sequencing and omics technologies with three-dimensional or even time-dependent four-dimensional techniques [104]. The discovery of more oocyte-specific proteins, involving oocyte-derived paracrine molecules and RBPs, will elucidate the mechanisms regulating oocyte meiosis. Integrated analyses of sequencing data, cross-species comparisons, and in vitro and in vivo experimentation using animal models might potentially unveil novel insights.

## Data Availability

Data sharing is not applicable to this review because no datasets were generated or analyzed.
